# Temporal trajectory of plasma miRNA during the peri-implantation stage: comparing implantation success and failure in single frozen–thawed blastocyst transfers

**DOI:** 10.3389/fendo.2026.1708664

**Published:** 2026-02-16

**Authors:** Lu Wang, Xiang Xiao, Hongya Yang, Ying Ju, Xiao He, Lingyin Kong, Shuqiang Chen, Xiuyu Feng, Jing Wu, Yue Liang, Yuan Ma, Juan Zhou, Bo Liang, Ni Jin, Jianlei Huang, Li Hai, Xiaohong Wang

**Affiliations:** 1 Department of Gynecology and Obstetrics, Tangdu Hospital, The Fourth Military Medical University, Xi’an, Shaanxi, China; 2 Basecare Medical Device Co., Research Department, Soochow, China; 3 School of Food and Biological Engineering, Jiangsu University, Zhenjiang, China; 4 School of Life Sciences and Biotechnology, Shanghai Jiao Tong University, State Key Laboratory of Microbial Metabolism, Joint International Research, Shanghai, China

**Keywords:** implantation failure, microRNAs, peri-implantation stage, peripheral blood, temporal trajectory

## Abstract

**Background:**

Implantation failure is the most common cause of pregnancy failure and is a major limiting factor in assisted reproduction. Plasma microRNA (miRNA) expression profiles show dynamic changes among individuals with successful implantation. However, the trajectory differences in plasma miRNA expression between women with implantation failure and success, as well as the potential role of those miRNAs, remain unclear.

**Methods:**

This study included 84 women who underwent single frozen–thawed blastocyst transfer in a natural cycle. For each patient, longitudinal plasma samples across five time points throughout the peri-implantation period (day0/D3/D5/D7/D9) were collected and underwent miRNA sequencing. The failure group (*n* = 27) encountered complete implantation failure, while the success group (*n* = 57) achieved a live birth. Using trajectory analysis and fuzzy c-means clustering, we identified dynamically differentially expressed (DDE) miRNAs and their dynamic expression patterns (DEPs) in a screening set (*n* = 52) and validated findings in an independent validation set (*n* = 32). Clinical correlations, functional annotation, prediction model, and *in vitro* validation of prioritized miRNA–target interactions were systematically conducted.

**Results:**

Twenty-four DDE miRNAs (FDR < 0.05) exhibited five temporal patterns: recession (R), growth (G), D3-trough (T), multimodal (M), and D5-trough (T2). The success group predominantly showed M-pattern miRNAs (62.5%), while failures demonstrated pattern transitions (M → T/T2) and exclusive T-pattern expression. Clinical relevance revealed eight DDE miRNAs associated with at least one clinical variable, with the majority being associated with estradiol/progesterone levels. Functional enrichment implicated Wnt/mTOR pathways in embryo implantation and decidualization. Six DDE miRNAs were successfully replicated in the validation set, while the support vector machine (SVM) model achieved area under the curve (AUC) values of 0.816 (D0) and 0.870 (D3). Additionally, the association between hsa-miR-214-3p and its target gene CTNNB1 was further confirmed in Ishikawa cells.

**Conclusions:**

Understanding the dynamic landscape changes of the miRNA transcriptome in individuals with implantation failure will help identify dynamic biomarkers from ovulation to the post-implantation stage, providing new insights into the pathological mechanisms of implantation failure and facilitating the research and development of new therapies in the clinical setting.

## Introduction

Implantation failure is a major limiting factor in assisted reproduction, accounting for over 72% of all *in vitro* fertilization (IVF) failures (1). Despite the rapid development of assisted reproductive technologies, the failure rate of embryo implantation has not seen a significant decrease, indicating an urgent need to elucidate the specific mechanisms underlying implantation. Implantation, a pivotal event for the establishment of a successful pregnancy, occurs within a confined time period, known as the “window of implantation” (WOI) (2, 3). During the peri-implantation period, the endometrium necessitates the synergistic action of ovarian hormones alongside cytokines, chemokines, lipids, and other signaling molecules to establish a microenvironment conducive to embryo implantation (4). In addition, the process involves various cell types, including luminal epithelial cells, glandular epithelial cells, stromal cells, and immune cells (5). Most importantly, this dynamic developmental process demands precise temporal regulation to ensure successful embryo implantation and subsequent pregnancy development.

MicroRNAs (miRNAs) are short, non-coding RNA molecules that are typically 18–25 nucleotides in length and function as posttranscriptional regulators of several gene targets (6). It is widely recognized that miRNAs play a significant role as key bioregulatory molecules in numerous physiological processes, including pregnancy (7). Due to ethical and technical constraints, it is challenging to obtain human endometrial tissues during the WOI, and animal models cannot fully substitute. Alternatively, peripheral blood miRNAs exhibit heightened sensitivity to overall physiological conditions and reflect the sophisticated interplay between the embryo and the maternal environment during the pivotal stages of implantation and early embryonic development. Previous studies have posited that miRNAs serve as potential regulatory factors in modulating endometrial receptivity in both human and mouse models (6, 8, 9). Nevertheless, previous studies discussing the association between miRNAs and the implantation process have largely been limited to single time-point investigations, which fail to fully delineate this dynamic process. Understanding the dynamic alteration of plasma miRNA landscaping of implantation failure may facilitate knowledge about the physiology of the implantation process and pathological status leading to implantation failure and further enhance the success of assisted reproductive techniques (10).

Consequently, our previous investigation first depicted the dynamic landscaping of plasma miRNA alterations during the peri-implantation stage among infertile women who underwent frozen–thawed embryo transfer (FET) with successful implantation and pregnancy, and we have pioneered the revelation that these dynamic plasma miRNA expression patterns may bear a significant correlation with endometrial receptivity (11). Despite these findings, the role of plasma miRNA expression patterns in the physiopathology of failed implantation remains largely uncharted. In the current study, we aimed to investigate and compare the dynamic alteration of plasma miRNA expression pattern between FET patients with successful implantation and those with implantation failure across various peri-implantation time points, specifically day 0 (D0, ovulation day), D3, D5, D7, and D9. It is our goal to identify dynamic differentially expressed (DDE) miRNAs and their dynamic expression patterns (DEPs), as well as provide insights into their potential role in the complex molecular dialogue that governs the establishment of a receptive endometrium, thereby influencing the outcome of embryo implantation. This comprehensive analysis is poised to enhance our understanding of the molecular determinants of implantation success and failure, offering new avenues for therapeutic intervention in reproductive medicine.

## Methods

### Study design and population

This work was carried out at the Center of Assisted Reproduction of Tangdu Hospital and wasapproved by the Ethics Committee of Tangdu Hospital, Air Force Medical University (TDLL-202210-15). All participants provided written informed consent. This study included infertile women who underwent a single FET during a natural cycle from October 2020 to June 2023. The subjects met the following inclusion criteria: maternal age ≦40 years and a regular menstrual cycle (21–35 days) with evidence of natural ovulation. The study enrolled infertile couples with tubal or male factor infertility, excluding those with endometriosis, adenomyosis, uterine abnormalities, intrauterine adhesions, hyperprolactinemia, thyroid disorders, or autoimmune diseases. A total of 84 participants were included: 57 of them experienced successful implantation and subsequently delivered a live birth, while 27 subjects encountered complete implantation failure. The enrolled participants were randomly allocated into a screening set and a validation set. The screening set comprised 18 patients with implantation failure and 34 with successful pregnancies, while the validation set included 9 patients with implantation failure and 23 with successful pregnancies (**Supplementary Figure S1**).

### Sample collection and small RNA sequencing

The protocol of sample collection, total RNA isolation and small RNA sequencing, and RNAsequencing data processing and mapping have been described elsewhere (11). In summary, all participants underwent follicular monitoring via transvaginal ultrasound and sex hormone testing prior to embryo transfer. Blood samples were collected for each patient at five different time points throughout the WOI, including D0, D3, D5, D7, and D9 in this cycle of embryo transfer. Day 0 was designated as the day of ovulation, which was assessed by a gynecologist based on plasma LH levels and the release of the dominant follicle (**Supplementary Figure S1**). For FET, all blastocysts were vitrified on day 5 or day 6 (calculated from the day of IVF) according to embryo development, and a single day-5 or day-6 blastocyst was thawed and transferred to the uterus on D5 (calculated from the day of ovulation), before D5 blood sampling. Peripheral blood samples were collected in 5-mL EDTA tubes and were centrifuged at 2,000×*g* for 10 min at 4°C, and the supernatants were obtained, aliquoted, labeled, and stored at −80°C until analysis.

Total RNA was isolated from 1 mL of plasma using the miRNeasy Serum/Plasma Advanced Kit (Cat # 217184, Qiagen, Dusseldorf, Germany) according to the manufacturer’s instructions. After extraction, RNA samples were eluted in 20 μL of nuclease-free water. cDNA was made for each sample using TruSeq^®^ Small RNA Library Prep Kit (Cat # RS-200-0024; Illumina, San Diego, CA, USA) based on the poly(A) RT-PCR method. After small RNA sequencing, the raw reads were quality-checked with a customized in-house pipeline. After adapter trimming/demultiplexing, reads were mapped to the reference genome (genome build GRCh 38; known sequences of miRNAs/piRNAs/other non-coding RNAs). Principal component analysis (PCA) was conducted to assess the presence of batch effects, given that the samples were processed in multiple batches. Potential outlier samples were identified using unsupervised analytical techniques, including PCA and *t*-distributed stochastic neighbor embedding (*t*-SNE).

### Trajectory analyses

In this study, our objective was to investigate the DDE miRNAs between the implantation failure and success groups through the lens of trajectory analysis. To tackle this, we employed the tradeSeq method, which is anchored in the negative binomial generalized additive model, for our analysis (12). This approach is well-suited for bulk time-course studies and is designed to infer trajectories that capture the dynamic fluctuations in gene expression over time. A total of 260 samples were screened, from 18 individuals in the implantation failure group and 34 individuals in the implantation success group (screening set), with each individual assessed at 5 time points. Three analytical modules were utilized to provide a comprehensive examination of differential expressions. Specifically, the Pattern Test module identifies miRNAs exhibiting distinct expression patterns over time by assessing whether the smoothed miRNA expressions are equivalent across time points between the two groups. The End Test module detects miRNAs with divergent expression endpoints by comparing the mean expression levels at the termini of group-specific smoothers. The Early Test module employs a statistical approach to identify a subset of miRNAs that demonstrate differential behavior at the initial stage between the two groups.

### Clustering of miRNAs according to their expression pattern

For DDE miRNAs identified through the aforementioned analyses, a logical next step is to cluster their expression patterns into DEPs. This approach provides a visual representation of the expression patterns and facilitates subsequent functional annotation. We employed the fuzzy c-means algorithm to group DDE miRNAs into different clusters based on their DEPs (13). Clustering for DDE miRNAs from both the implantation success and failure groups was performed independently.

### Relevance to clinical traits

To address the relevance of identified DE miRNAs to clinical traits, the linear mixed model was utilized. The linear mixed model was employed to account for repeated measures nested within individuals. Baseline information (female age and BMI), basal hormone levels (including FSH, LH), anti-Müllerian hormone (AMH) level, antral follicle count (AFC), number of oocytes, dynamic E2, and progesterone (P) were analyzed. A *P*-value of ≤0.05 was deemed significant.

### MicroRNA target prediction

To predict target genes of candidate differential miRNAs, experimentally verified miRNA–target gene interaction databases including miRecords, mirTarBase, and TarBase v9.0 were searched (14–16). In order to further improve the credibility of target genes, target genes verified by Western blot, Luciferase reporter assay, and qRT-PCR were selected as candidate miRNA–target genes. The regulatory network between the miRNAs and their targets was constructed and visualized using Cytoscape (17).

### Functional enrichment analysis

Gene Ontology (18), Kyoto Encyclopedia of Genes and Genomes (19), Reactome gene sets (20), and WikiPathways (21) enrichment analysis of predictive gene signatures were performed using Metascape (22). Enrichment *P*-values were adjusted using Benjamini–Hochberg correction, and an adjusted *P*-value of ≤0.05 was used as the significance cutoff.

### Assessment of miRNA expression in the validation set

To assess the robustness of identified DDE miRNAs, we performed association tests in an independent validation set. The validation set included 115 samples (23 individuals with five time points) in the implantation success group and 95 samples (9 individuals with five time points) in the implantation failure group. DDE miRNAs were examined utilizing the Wilcoxon rank-sum test between the two groups at each time point. DDE miRNAs that exhibited the same directional effects as those observed in the screening set and were also found to be significant in the validation set were considered to be replicative DDE miRNAs.

### Prediction of implantation outcome using early DDE miRNAs

Early DDE miRNAs refer to miRNAs identified by the early test that showed different expression patterns at the beginning stage, namely, D0 and D3. Establishing the prediction model using peripheral blood miRNAs before embryo implantation would provide useful clinical insights and benefit clinical therapy. The support vector machine (SVM) was established as a prediction model using replicative early DDE miRNAs as predictors. SVM is a binary classification supervised machine learning algorithm. Its core principle is to identify an optimal boundary that separates different data categories while maximizing the margin between this boundary and the nearest data points. The expression levels of the specified early DDE miRNAs (from D0 or D3) were used as input features to generate a probability score; samples with a probability ≥0.5 were classified as predicted implantation success. Internal 10-fold cross-validation was employed to tune hyperparameters and prevent overfitting. Model performance was evaluated using the area under the curve (AUC) in an independent validation set.

### Function of DDE miRNA in endometrial epithelial cell lines

Having observed the validated DDE miRNAs in human plasma, we proceeded to explore the function of prioritized miRNAs in the endometrial epithelial cells. During the peri-implantation stage, changes in miRNA expression in endometrial epithelial cells mirror the coordinated processes of embryo positioning and adhesion, which are intimately linked to endometrial receptivity and implantation outcome (23). Hence, we validated the function of prioritized miRNAs in human Ishikawa cell lines. Ishikawa cells, derived from human endometrial carcinoma, are widely employed as an *in vitro* model for studying embryo implantation (24). These well-differentiated cells maintain functionality and serve as a useful model to study embryo implantation.

### Cell culture

Human Ishikawa cell lines were obtained from the American Type Culture Collection (ATCC). Ishikawa cells were cultured at 37°C in a 5% CO_2_ environment in Dulbecco’s modified Eagle’s medium (DMEM)/Ham’s F12 medium with L-glutamine and 15 mM of HEPES (10092-CVRC, Corning NY, USA) with 10% fetal bovine serum (10099141C-1, Gibco Australia).

### Cell transfection

The miRNA mimic for hsaicfec214‐3p-agomir and negative control (NC) mimics were purchased from Qingke Biotech Co., China. These miRNAs were transfected into cells using Lipofectamine 2000 (11668019, Thermo Fisher Waltham, MA, USA). Transfections of miRNA were performed at a concentration of 20 μM on 6-well plates of human Ishikawa cells at 60%−70% confluence, 24 h after plating, according to the manufacturer’s protocol. After 6 h of culture, the cell-transfected culture medium was replaced with fresh medium. The cells were collected for subsequent experiments after 48 h of incubation. The efficiency of transfection was determined by real‐time polymerase chain reaction (RT-qPCR).

### Real-time quantitative reverse transcription–polymerase chain reaction

Total RNA was extracted from cell lines using TRIzol reagent (15596018, Life, USA), followed by RT using PrimeScript First-Strand cDNA Synthesis kit (RR036A, TaKaRa, Japan), according to the manufacturer’s protocols. The mRNA expression levels of CTNNB1 were evaluated by PCR with a Bio‐Rad CFX96 real‐time PCR instrument (Bio‐Rad) and TB Green™ Advantage^®^ qPCR Premix for Q‐PCR according to the manufacturer’s protocol (TaKaRa, 639676). The RT−qPCR primers used were as follows: CTNNB1, forward 5′-AGCTTCCAGACACGCTATCAT-3′, reverse 5′-CGGTACAACGAGCTGTTTCTAC-3′; and β-actin, forward 5′-CGTCACCAACTGGGACGACA-3′ and reverse 5′-CTTCTCGCGGTTGGCCTTGG-3′. β-Actin was employed as an endogenous control. The samples were analyzed using the 2^−ΔΔCt^ method to analyze the relative mRNA expression level. Experiments for each analysis were run in triplicate. Values are expressed as the mean of 2^−ΔCt^ ± standard deviation.

### Western blot

Cells were lysed on ice in tissue protein extraction reagent (78510, Thermo, USA) with proteinase inhibitors (HY-K0010, MCE, USA) and phosphatase inhibitors (4906837001, Roche, Switzerland). The protein lysate was placed on ice for 20 min and centrifuged at 14,000 rpm for 20 min at 4°C. The extracted supernatants were then harvested for subsequent testing. The protein concentration was detected using a BCA protein assay kit (23227, Thermo, USA). Proteins were separated using sodium dodecyl sulfate Tris-glycine gels (Bio-Rad, USA), transferred onto PVDF membranes, blocked in 5% non-fat milk, and hybridized overnight at 4°C with primary antibodies. The primary antibodies included CTNNB1 (1:5,000, 51067-2-AP, Proteintech, China) and β-actin (1:1,000, 4970S, CST, USA). The secondary antibody that we used was HRP-labeled goat anti-rabbit IgG (1:3,000, 7074P2, CST, USA). The membranes were washed with Tris-buffered saline containing 0.01% Tween-20. Protein bands were visualized using Immobilon Chemiluminescence Reagent (WBKLS0500, Millipore, USA) and analyzed using a ChemiDox Gel imaging system (Bio-Rad).

### Coding

All analyses in this study were performed using R 4.0.2 software (R Foundation for Statistical Computing, Vienna, Austria). In different stages, packages including “sva,” “TradeSeq,” “Mfuzz,” and “multiMiR” were used. In trajectory analysis, dynamically differentially expressed miRNAs were selected based on the following criteria: |log_2_ fold change| >0.5 and FDR-adjusted *P* < 0.05. Otherwise, *P <*0.05 was considered to have suggestive significance. For the SVM model, the R package “e1071” was used, and the radial basis function kernel was selected.

## Results

### Clinical baseline characteristics

As depicted in the flowchart (**Supplementary Figure S1**), a total of 170 plasma samples were collected from 34 women who experienced successful implantation, and 90 plasma samples were collected from 18 women who experienced implantation failure (screening set), across five distinct time points during the peri-implantation stage. The basic clinical characteristics are presented in **Supplementary Table S1**. The information regarding the implantation success group has been presented in our previous study (11). Generally, no significant difference was observed between the implantation failure and success groups, including female and male age, BMI, blood pressure, infertile duration and type, basal sex hormone (FSH, LH, E2), AMH, AFC, number of oocytes retrieved and embryos, and endometrial thickness on D0. The mean age was 30.62 years in the implantation failure group and 31.17 years in the success group (*P* = 0.476). After treatment, women in the implantation failure group had a mean of 13 oocytes and 9 embryos. Those in the success group had 14 oocytes and 10.4 embryos (*P* > 0.05). The peaks of estradiol (E2) and progesterone (P) levels occurred around implantation day and gradually decreased in pace with the failure of implantation (**Supplementary Figure S2**). Significant differences in E2 and P levels were observed between the implantation success and failure groups at D9 (**Supplementary Figure S2**).

### Quality of sequencing data and microRNAs with different expression patterns between the implantation failure and success groups

A total of 2,656 miRNAs with valid read counts were identified before filtering. Quality assessment was performed using unsupervised learning methods in the combined groups, and batch effects were corrected with the ComBat-seq method (**Supplementary Figure S3**). To conduct a comprehensive analysis of the differences between the implantation failure and success groups, three analytical modules were utilized. A total of 24 miRNAs were identified as dynamically differentially expressed between the two groups (**Table 1**; **Supplementary Tables S2–4**). Specifically, the Pattern Test module identified 20 miRNAs exhibiting DEPs between the two groups. The End Test module revealed 17 miRNAs with divergent expression at endpoints, while the Early Test module detected 15 miRNAs with differential expression at the initial stage between the two groups. While the majority of miRNAs were found to overlap across various analysis modules, two miRNAs, hsa-miR-1249-3p and hsa-miR-370-3p, were uniquely identified in the Pattern Test. Similarly, the End Test exclusively identified hsa-miR-1260a and hsa-miR-31-5p, whereas the Early Test specifically recognized hsa-miR-127-3p and hsa-miR-1323.

**Table 1:** Dynamic differentially expressed miRNAs identified by trajectory analysis.

MicroRNA	Pattern test	End test	Early test
hsa-miR-122b-3p	DE	Not DE	DE
hsa-miR-1249-3p	DE	Not DE	Not DE
hsa-miR-125b-5p	DE	DE	Not DE
hsa-miR-125b-1-3p	DE	DE	DE
hsa-miR-1307-5p	DE	DE	DE
hsa-miR-134-3p	DE	Not DE	DE
hsa-miR-149-5p	DE	DE	Not DE
hsa-miR-195-5p	DE	Not DE	DE
hsa-miR-214-3p	DE	DE	DE
hsa-miR-299-5p	DE	DE	Not DE
hsa-miR-34b-3p	DE	DE	DE
hsa-miR-34c-5p	DE	DE	DE
hsa-miR-34c-3p	DE	DE	Not DE
hsa-miR-370-3p	DE	Not DE	Not DE
hsa-miR-4433b-3p	DE	DE	DE
hsa-miR-4749-5p	DE	DE	DE
hsa-miR-5683	DE	DE	DE
hsa-miR-665	DE	DE	DE
hsa-miR-877-3p	DE	DE	Not DE
hsa-miR-99b-5p	DE	DE	DE
hsa-miR-1260a	Not DE	DE	Not DE
hsa-miR-31-5p	Not DE	DE	Not DE
hsa-miR-127-3p	Not DE	Not DE	DE
hsa-miR-1323	Not DE	Not DE	DE

To illustrate the dynamic expression patterns of DDE miRNAs between the two groups, clustering analysis was conducted independently for the 24 miRNAs in each group. A detailed classification of 24 DDE miRNAs in each group is listed in **Table 2**. In general, the DDE miRNAs were categorized into five DEPs: recession (R), growth (G), trough with turning point around D3 (T), multimodal (M), and trough with turning point around D5 (T2) (**Figure 1**). For the T pattern, the turning point occurred around D3 and was exclusively observed in the failure group. For the T2 pattern, the turning point occurred around D5, the embryo transfer day. In the implantation success group, the majority of DDE miRNAs conform to the M pattern, characterized by a peak value around the day of embryo transfer, with troughs occurring before (around D0) and after (around D7). However, the majority of DDE miRNAs exhibiting the M pattern in the success group transitioned into the G, T, and T2 patterns in the failure group. Relative to the M pattern observed in the successful implantation group—characterized by a peak on day 5—both the T and T2 patterns in the implantation failure group represent a flattening or loss of this peak, indicating that the same miRNAs remain significantly lower on day 5. This suggests that the transition in their expression patterns may be related to a shifted implantation window. Correspondingly, most of the miRNAs in the T cluster and all miRNAs belonging to the T2 cluster in the failure group were derived from the M cluster in the success group. Likewise, pattern transformations occurred from R to G and T, from G to R, and from T2 to R. The above results indicate that not only are there DDE miRNAs between the success and failure groups, but also a transformation in DEPs has occurred.

**Table 2:** Fuzzy c-means clustering results of 24 dynamic differentially expressed miRNAs.

miRNA	DEP in success group	Membership in success group	DEP in failure group	Membership in failure group
hsa-miR-122b-3p	G	0.363	R	0.991
hsa-miR-125b-1-3p	M	0.993	T2	0.445
hsa-miR-127-3p	M	0.995	M	0.792
hsa-miR-1307-5p	G	0.994	G	0.994
hsa-miR-1323	M	0.692	T	0.619
hsa-miR-134-3p	G	0.997	G	0.990
hsa-miR-195-5p	T2	0.955	R	0.536
hsa-miR-214-3p	M	0.999	T	0.755
hsa-miR-34b-3p	M	0.990	M	0.771
hsa-miR-34c-5p	M	0.996	T	0.778
hsa-miR-4433b-3p	R	0.989	G	0.994
hsa-miR-4749-5p	R	0.991	G	0.995
hsa-miR-5683	R	0.871	T	0.576
hsa-miR-665	R	0.988	G	0.994
hsa-miR-99b-5p	M	0.998	T2	0.560
hsa-miR-125b-5p	M	0.992	T2	0.936
hsa-miR-1260a	M	0.883	T2	0.755
hsa-miR-149-5p	M	0.996	T2	0.810
hsa-miR-299-5p	M	0.989	G	0.520
hsa-miR-31-5p	M	0.997	T	0.907
hsa-miR-34c-3p	M	0.961	G	0.540
hsa-miR-877-3p	M	0.965	G	0.681
hsa-miR-1249-3p	M	0.997	T	0.950
hsa-miR-370-3p	G	0.274	G	0.935

#Distinct expression pattrens (DEPs) are defined as recession (R), growth (G), trough with turning point around D3 (T), multi-modal (M), and trough with turning point around D5 (T2).

**Figure 1:**
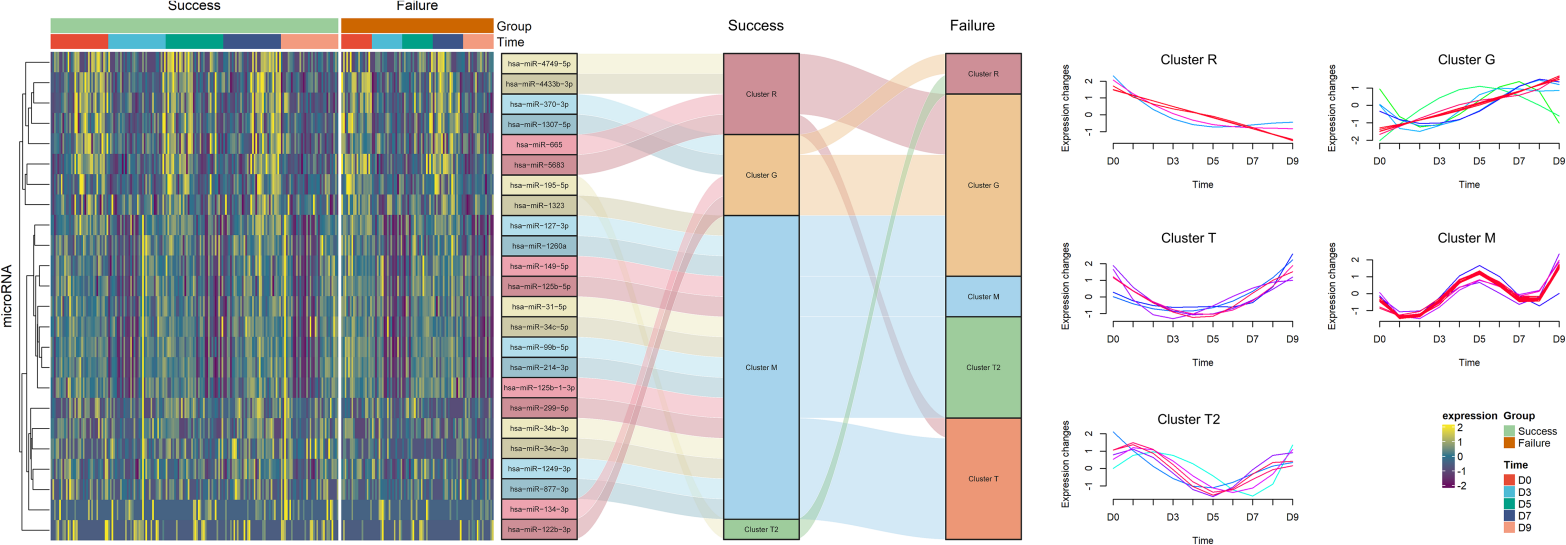
Overview of dynamic differential expression profiles between the implantation failure and success groups. Left panel: Heatmap plot represents the 24 dynamic differentially expressed (DDE) microRNAs (miRNAs) between the implantation failure and success groups. Middle panel: Sankey diagram illustrates the dynamic expression patterns (DEPs) of DDE miRNAs and their distribution in the implantation success and failure groups. The right panel shows five DEPs of miRNA expression identified by fuzzy c-means clustering. R, recession; G, growth; M, multimodal; T, trough with turning point around D3; T2, trough with turning point around D5.

### Relevance with clinical traits

**Figure 2:**
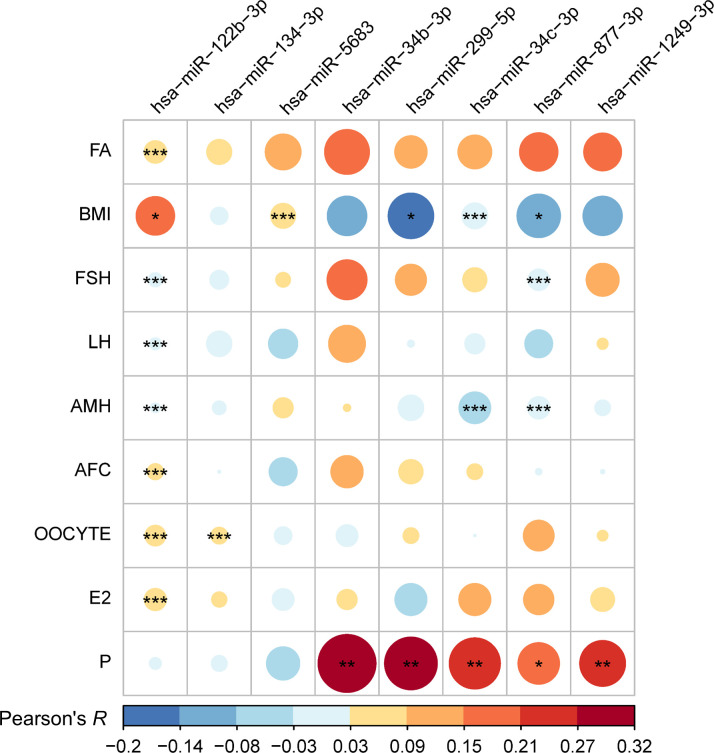
Correlation matrix plot showing associations between dynamic differentially expressed miRNAs and clinical traits. Eight dynamic differentially expressed (DDE) miRNAs with at least one significant trait using the linear mixed model are shown. The color gradient indicates the direction, i.e., positive (red) and negative (blue), and the strength of the correlation. The significance of *P*-values was labeled as follows: * 0.01 < *P*-value < 0.05, ** 0.001 < *P*-value < 0.01, *** *P*-value < 0.001. FA, female age; AMH, anti-Müllerian hormone; AFC, antral follicle counting; ET, endometrial thickness; E2, estradiol; P, progesterone.

As shown in **Figure 2**, a total of eight DDE miRNAs demonstrated significant associations with at least one clinical variable. In general, most of the DDE miRNAs (except for hsa-miR-134-3p and hsa-miR-5683) were associated with the levels of E2 or P, suggesting that they may serve as sensitive indicators of plasma endocrine levels.

Regarding their DEPs, we observed that DDE miRNAs within the same DEP tend to exhibit similar associations with clinical traits (**Table 3**). Specifically, DEPs of miRNAs in the success group were predominantly distributed in the M pattern, exhibiting a positive association with P levels. For DDE miRNAs transitioning from the M to G pattern, an additional negative association with BMI was observed. In the case of DDE miRNAs with consistent G to G, transition G to R, and R to T patterns, positive correlations with BMI or the number of oocytes were noted. In summary, the M pattern is characterized by fluctuating changes during the peri-implantation period, and miRNAs within this pattern are associated with indicators that present a cycle-dependent manner, such as progesterone levels. Conversely, the G or R pattern shows linear changes during the peri-implantation period, and miRNAs associated with these patterns are related to relatively stable physiological indicators, such as BMI or basal levels of sex hormones.

**Table 3:** Clinical relevance of dynamic differentially expression miRNAs.

MicroRNA	DEP in success group	DEP in failure group	Clinical relevance
hsa-miR-122b-3p	G	R	Positively correlated with age, BMI, AFC, OOCYTE and E2, negatively correlated with FSH, LH and AMH
hsa-miR-134-3p	G	G	Positively correlated with OOCYTE
hsa-miR-5683	R	T	Positively correlated with BMI
hsa-miR-34b-3p	M	M	Positively correlated with P
hsa-miR-299-5p	M	G	Positively correlated with P, negatively correlated with BMI
hsa-miR-34c-3p	M	G	
hsa-miR-877-3p	M	G	
hsa-miR-1249-3p	M	T	Positively correlated with P

#Distinct expression pattrens (DEPs) are defined as recession (R), growth (G), trough with turning point around D3 (T), multi-modal (M), and trough with turning point around D5 (T2).

### Target gene prediction of DDE miRNAs

**Figure 3:**
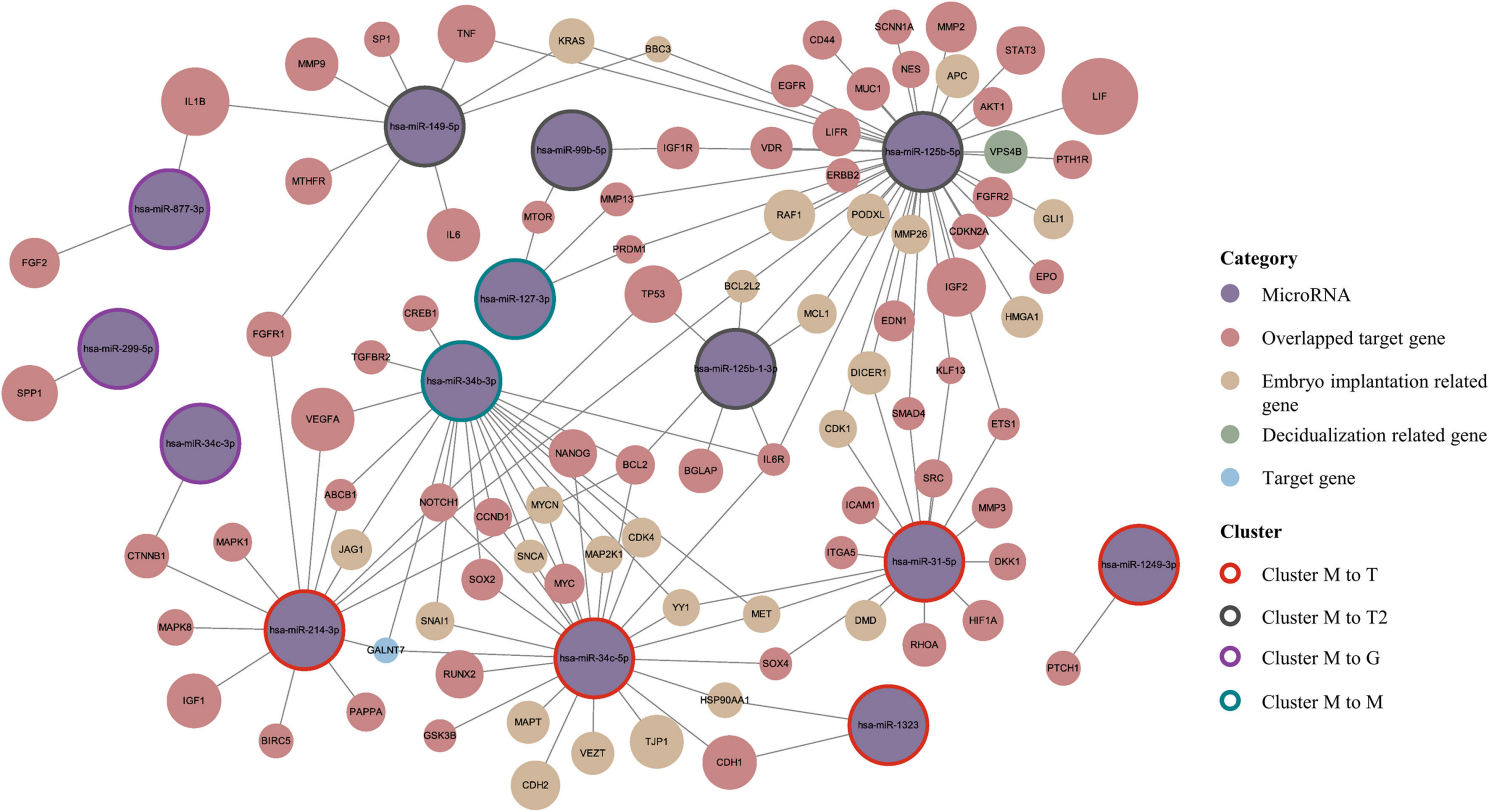
Regulatory miRNA–mRNA networks for dynamic differentially expressed miRNAs belonging to the M pattern in the implantation success group. Due to limited space, targets were filtered with a cutoff of relevance score above 3 or common targets were predicted by at least two miRNAs. The full list of predicted targets for miRNAs can be found in **Supplementary Table S5**.

For all DDE miRNAs, their target genes were predicted using the miRecords, mirTarBase, and TarBase v9.0 databases. Furthermore, we examined whether those target genes were involved in the process of endometrium decidualization and embryo implantation. This information was downloaded from the GeneCards database (https://www.genecards.org/) by searching key words (decidualization or embryo implantation). We searched all the above 24 DDE miRNAs and found that 20 of them had available target genes (**Supplementary Table S5**). **Figure 3** demonstrates the prediction network of DDE miRNAs belonging to the M pattern in the successful implantation group. It indicates that their predicted target genes exhibit high connectivity or strong association with embryo implantation or decidualization, as evidenced by relevance scores exceeding 3. Specifically, transitions from the M pattern to the T or T2 pattern are postulated to be associated with a shifted implantation window, thereby potentially influencing endometrial receptivity. Consistently, target genes related to decidualization and embryo implantation predominantly fall under the regulation of miRNAs within these DEPs. Key genes, including VEGFA, IGF1, CTNNB1, NOTCH1, and MAPK1, possess relatively strong evidence supporting their associations with both decidualization and embryo implantation and are regulated by miRNAs that belong to cluster M to T. Similarly, miRNAs in cluster M to T2 target essential genes including LIF, MTOR, IL6, IL1B, and MMP2, thereby underscoring the potential role of the identified miRNAs. Furthermore, other DDE miRNAs with DEP transitions between the implantation success and failure groups also warrant attention. The regulatory network of hsa-miR-665 belonging to the pattern R to G and hsa-miR-195-5p belonging to the pattern T2 to R were also investigated (**Supplementary Figure S4**). A large part of the target genes are associated with embryo implantation or with both processes, including SMAD3, VEGFA, FGF2, RUNX2, WNT7A, and BCL2.

### Functional enrichment for predicted target genes

**Figure 4:**
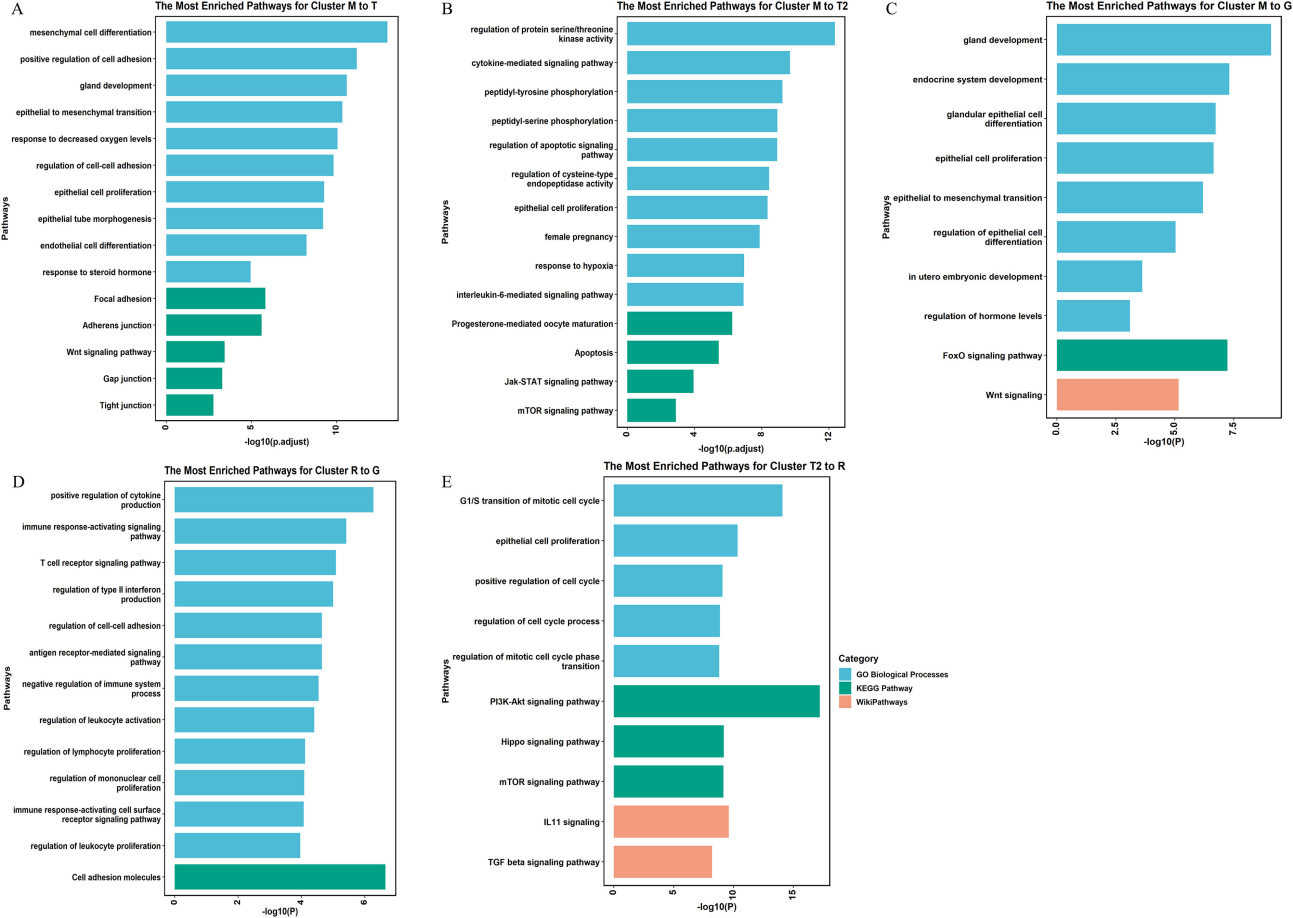
Functional enriched signaling pathway. Gene Ontology (GO) biological process, Kyoto Encyclopedia of Genes and Genomes (KEGG) pathway, and WikiPathway terms are searched and enriched by Metascape. **(A–E)** The most enriched pathways for the predicted targets of differentially expressed miRNAs in cluster M to T **(A)**, cluster M to T2 **(B)**, cluster M to G **(C)**, cluster R to G **(D)**, and cluster T2 to R **(E)**. All bars represent log_10_-transformed adjusted *P*-values. R, recession; G, growth; M, multimodal; T, trough with turning point around D3; T2, trough with turning point around D5.

Functional enrichment analysis was conducted to elucidate the biological functions of the identified DDE miRNAs and to gain insights into their association with the process of embryo implantation. We specifically focused on the functional annotation of DDE miRNAs with DEP transitions between the implantation success and failure groups, which may be indicative of signaling pathways related to aberrant implantation events. The T pattern was exclusively expressed in the implantation failure group and, therefore, may be indicative of signaling pathways associated with complete implantation failure. The functional annotation for the transition from pattern M to T highlighted cell junctions (**Figure 4A**), specifically “adherens junction,” “gap junction,” and “tight junction,” which are essential for facilitating endometrial receptivity and establishing an optimal environment for embryo implantation. Moreover, **Figure 4B** displays the enriched biological pathways for pattern M to T2. Pathways related to cytokine signal transduction, including “peptidyl-tyrosine phosphorylation,” “peptidyl-serine phosphorylation,” “interleukin-6 mediated signaling pathway,” and “Jak-STAT signaling pathway,” were significantly enriched. Furthermore, in the transition from the M pattern to the G pattern (**Figure 4C**), multiple biological processes and signaling pathways crucial for decidualization were significantly enriched, including “epithelial cell proliferation,” “mesenchymal–epithelial transition,” and FoxO and Wnt signaling pathways. Biological processes related to the endocrine system were also enriched, such as “gland development,” “endocrine system development,” and “regulation of hormone levels.” These pathways play a crucial role in the process of embryo–uterine crosstalk. Additionally, hormone-related signaling pathways were observed for all patterns belonging to the M pattern in the success group, consistent with their clinical relevance with P levels.

The transition from the R pattern to the G pattern exhibits a completely opposite trend of change during the peri-implantation period. Consistent with the non-fluctuating characteristics of the DEP, enrichment analysis reveals that the target genes of miRNAs associated with this pattern are predominantly enriched in immune-related signaling pathways, including the “immune response-activating signaling pathway,” “T-cell receptor signaling pathway,” and “regulation of type II interferon production” (**Figure 4D**). Similarly, the functional enrichment for pattern T2 to R emphasizes significant signaling pathways, including PI3K-Akt, Hippo, mTOR, IL11, and TGF-beta (**Figure 4E**).

### Assessment of miRNA expression in the validation set

The baseline characteristics are detailed in **Supplementary Table S6**. A total of six miRNAs—hsa-miR-125b-1-3p, hsa-miR-1323, hsa-miR-195-5p, hsa-miR-214-3p, hsa-miR-4433b-3p, and hsa-miR-99b-5p—were identified as replicative DDE miRNAs (**Supplementary Figure S5**). We found that the temporal expression differences detected in the validation set were highly consistent with the dynamic expression patterns identified in the screening set. For example, hsa-miR-125b-1-3p exhibited an M pattern in the success group and a T2 pattern in the failure group in the screening set; in the validation set, we again observed higher expression at D5 than at D9 in the success group (*P* = 0.065), whereas no such change was present in the failure group. Similarly, hsa-miR-195-5p displayed a T2 pattern in the success group and an R pattern in the failure group in the screening set, and the validation set revealed the same directional trend. Notably, the differential expression on D3 was replicated for hsa-miR-125b-1-3p and hsa-miR-195-5p, and both were upregulated in the failure group. Similarly, upregulation was kept on D0 and D3 for hsa-miR-214-3p and on D9 for hsa-miR-4433b-3p. The upregulation between the success and failure groups was additionally observed across all five time points for hsa-miR-1323 and hsa-miR-99b-5p.

### Function of DDE miRNAs in endometrial epithelial cell lines

**Figure 5:**
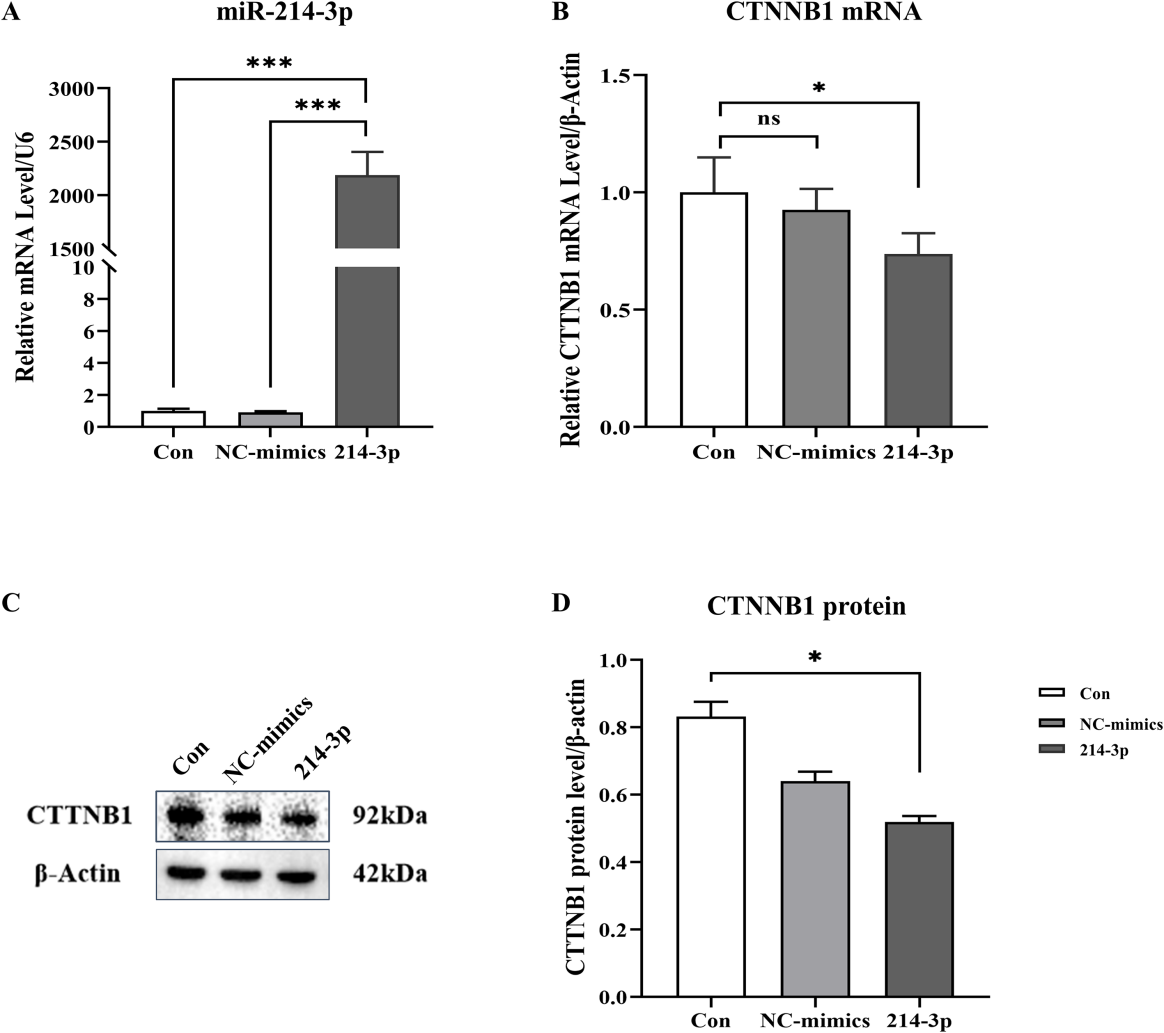
Cell transfection of miR‐214‐3p and CTNNB1 expression. **(A)** miR-214-3p expression in Ishikawa cell lines transfected with hsa‐miR‐214‐3p for 48 h, normalized to U6, as detected by RT-qPCR. **(B)** CTNNB1 mRNA expression in Ishikawa cell lines transfected with hsa-miR-214-3p for 48 h, normalized to β-actin, as detected by RT-qPCR. **(C, D)** CTNNB1 protein levels in Ishikawa cell lines transfected with hsa‐miR‐214‐3p for 48 h (normalized with β-actin, *n* = 3). Data are expressed as means ± SEM; CON, control; NC, negative control. *P-value <0.05 compared with controls; ***P-value <0.001 compared with controls.

Candidate miRNAs for *in vitro* cellular validation were selected from the six reproducible DDE miRNAs. Among these, hsa-miR-214-3p was chosen because it has the largest number of predicted targets and exhibits an expression pattern specific to the implantation failure group, suggesting a potential association with altered endometrial receptivity during the implantation window. The Wnt/β-catenin (CTNNB1) signaling pathway is essential for the process of embryo implantation, coordinating the interactions between the uterus and the embryo (25). CTNNB1 is a putative target (downregulated) of hsa‐miR‐214‐3p (upregulated, **Supplementary Figure S5D**). Therefore, we aimed to validate the coexpression of hsa‐miR‐214‐3p and CTNNB1 in Ishikawa cells. Firstly, we confirmed transfection efficiency using RT-qPCR after transfection of hsa-miR-214-3p agomir into Ishikawa cells and found significantly increased hsa‐miR-214-3p compared with the controls (**Figure 5A**). It was found that overexpression of hsa‐miR‐214‐3p by agomir significantly decreased the mRNA expression of *CTNNB1* (**Figure 5B**). Similarly, overexpression of hsa-miR-214-3p by agomir significantly decreased the protein levels of CTNNB1 compared with the control group (**Figures 5C, D**).

### Prediction of implantation outcome using early DDE miRNAs

**Figure 6:**
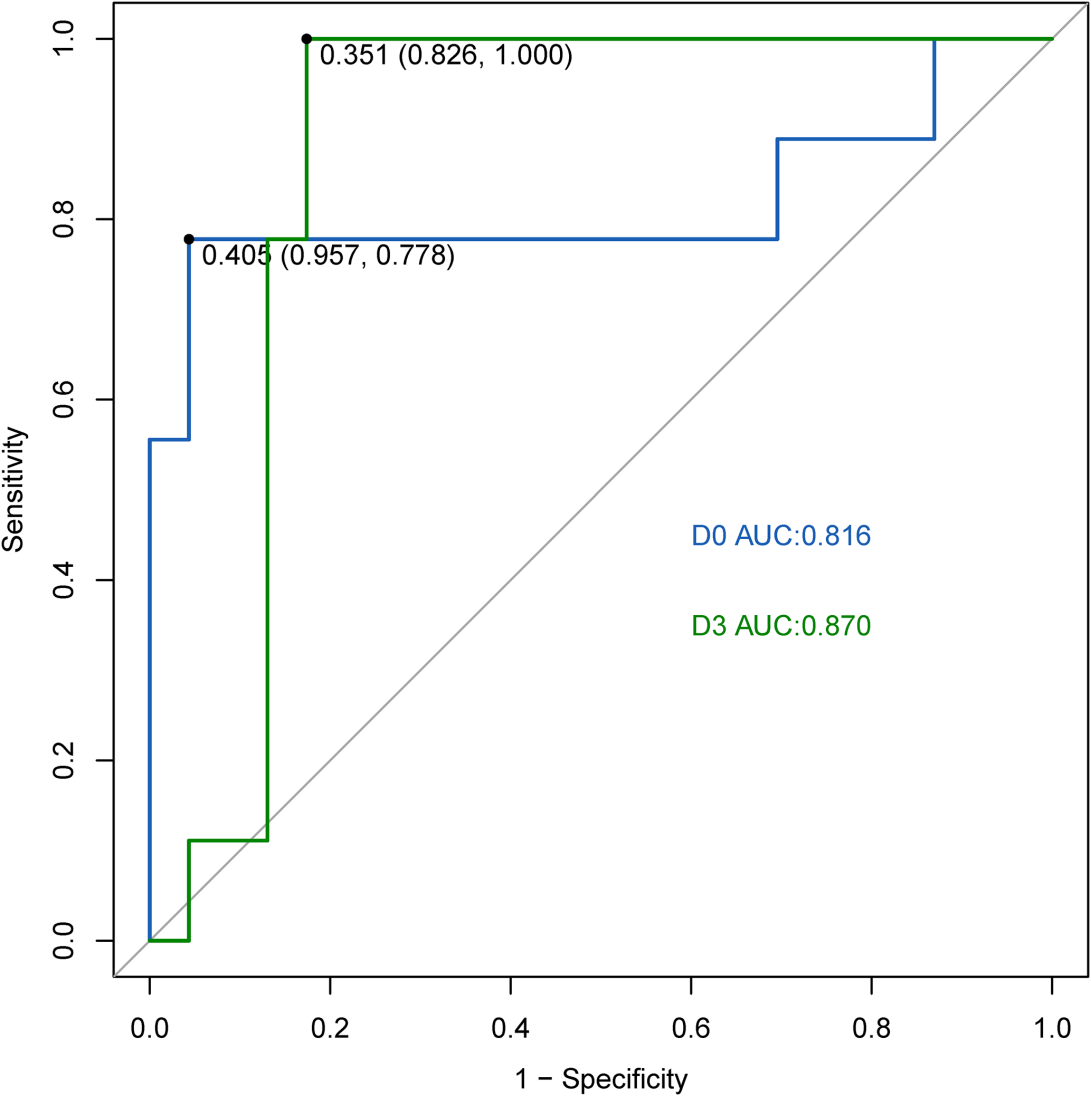
ROC curve analysis of the SVM model for predicting the implantation outcome of infertile women on day 0 and day 3 in the validation set.

Among the 15 early DDE miRNAs, three (hsa-miR-1323, hsa-miR-99b-5p, and hsa-miR-214-3p) were replicative DDE miRNAs on D0, while five (hsa-miR-1323, hsa-miR-99b-5p, hsa-miR-214-3p, hsa-miR-125b-1-3p, and hsa-miR-195-5p) were replicative on D3. On D0, the AUC value of the SVM model was 0.816 (**Figure 6**). The specificity and sensitivity were 0.957 and 0.778, respectively. On D3, the AUC value of the SVM model was 0.870 (**Figure 6**). The specificity and sensitivity were 0.826 and 1, respectively.

## Discussion

The variable miRNA expression profiles observed at different endometrial stages suggest that miRNAs play a regulatory role in endometrial receptivity in both human and mice (26–28). Circulating miRNAs are considered promising biomarkers due to their stability, rich biological information content, and the non-invasive nature of their detection. The associations between circulating miRNA expression levels and pregnancy have long been discussed (29–32). Moreover, previous studies have explored the potential role of circulating miRNAs in embryo implantation (33, 34). However, it should be noted that embryo implantation is a dynamic process. Previous studies often employed study designs with a single time point or treated data from multiple time points as independent data sets, both of which can lead to an inability to fully capture the dynamic characteristics. In the current study, trajectory analyses were conducted to integrate data from five time points, thereby identifying a total of 24 DDE miRNAs and 5 DEPs.

Suboptimal endometrial receptivity appears to be a major cause of implantation failure. Consistently, we found that the pattern transition from M to T and M to T2 may be related to the shifted WOI. In line with this hypothesis, miRNAs belonging to these patterns were found to be associated with endometrial receptivity in various studies. For instance, miR-125b has been shown to inhibit cell migration and invasion in endometrial epithelial cells, thereby regulating endometrial receptivity (35). miR-149 has been shown to regulate endometrial receptivity and the trophoblast attachment process by targeting PARP-2 (36). Moreover, Al-Rubaye S et al. identified that the expression levels of blood miR-214-3p in the M to T pattern were significantly higher in patients with recurrent pregnancy loss of unknown causes compared to controls (37), suggesting that miR-214-3p may play a role in regulating endometrial receptivity. In addition, high expression of miR-34c was found in the caprine receptive endometrium, where it functions to induce endometrial epithelial cell apoptosis via the RAS/RAF/MEK/ERK and PI3K/AKT/mTOR pathways (38). Dysregulation of the miR-34 families has been discussed in the endometrium of women with endometriosis, which is associated with a delayed transition from the proliferative to the secretory phase (39). Similarly, our results identified the M pattern (with a peak value on day 5) of miR-34c-3p and miR-34c-5p in the implantation success group and a pattern transition to the G or T pattern in the implantation failure group. Furthermore, hsa-miR-99b-5p in human endometrial fluid was considered a non-invasive biomarker for receptive endometrium (40). An elevation in miR-31 levels during the WOI was observed in both the human endometrium and serum, suggesting that miR-31 may serve as a potential biomarker for optimal endometrial receptivity (33).

The collaboration between estrogen and progesterone plays a crucial role in endometrial receptivity through the initiation of paracrine or autocrine signaling (41). Clinical relevance analysis identified one DDE miRNA (hsa-miR-122b-3p) associated with E2 levels. A regulatory role of estrogen on miR-122 has been reported in zebrafish and rat (42, 43). In human hepatocellular carcinoma cells, treatment with estrogen results in the upregulation of miR-122 and induces apoptosis (7), findings that are consistent with our observation of a sustained upward trend within the success group, characterized by the G pattern. Among the five DDE miRNAs associated with P levels, two miRNAs were members of the miR-34 family (hsa-miR-34b-3p and hsa-miR-34c-3p). Dysregulation of the miR-34 miRNA families was observed for endometriosis patients with progesterone resistance (39). Similarly, associations between miR-299 and miR-877-3p with sex hormone have been reported (44, 45), suggesting that they may participate in the process of implantation through the endocrine-related pathway.

Functional annotation of identified DDE miRNAs also supported their role in the process of decidualization and embryo implantation. For instance, “Wnt signaling” and “mTOR signaling pathway” were significantly enriched across different DEPs. Wnt signaling is suggested to be critical for endometrial changes related to implantation such as decidualization and endometrial gland formation (46). Results from multiple animal studies suggest that the mTOR signaling pathway is crucial for maintaining successful implantation and early embryonic development (47, 48). Furthermore, the most enriched biological processes and signaling pathways include “epithelial to mesenchymal transition,” “*in utero* embryonic development,” and “response to hypoxia,” as well as FoxO, TGF-beta, IL-6, and PI3K-AKT signaling pathways. These signaling pathways are considered to be crucial for embryonic development and implantation (49–51). In addition, the most enriched pathways for the transition from pattern R to G encompass immune-related pathways, suggesting that these miRNAs may modulate maternal immune tolerance by targeting certain key genes.

Wnt signaling regulates embryo implantation, and the ablation of specific Wnt signaling components leads to infertility (25, 52). The canonical Wnt pathway triggers gene expression mediated by CTNNB1, which regulates the endometrial cell function and embryo implantation (53, 54). Due to the important role of CTNNB1 in embryo implantation, we also investigated whether hsa-miR-214-3p, one of the DDE miRNAs, regulated the expression of CTNNB1. It was found that overexpression of hsa-miR-214-3p significantly downregulated the protein levels of CTNNB1, which indicated that these DDE miRNAs may play a critical regulatory role in the process of embryo implantation.

Although differential analysis based on pattern expression is presented, this study employs an observational design and can only suggest associations rather than establish causality. Nevertheless, evidence for an association derived from time-series analysis is considered methodologically stronger than that which would be obtained from a case–control study, as per the hierarchy of evidence proposed by the Oxford Centre for Evidence-Based Medicine (https://www.cebm.ox.ac.uk/resources/levels-of-evidence/ocebm-levels-of-evidence). In addition, the findings of this study are derived from a specific population undergoing natural cycle single frozen–thawed blastocyst transfer. The applicability of our findings to hormone replacement therapy (HRT) cycles or ovarian stimulation cycles remains to be validated, since exogenous hormones may alter miRNA profiles. The sample sizes between the implantation success (*n* = 57) and failure (*n* = 27) groups were imbalanced, reflecting the natural clinical distribution in our setting. The potential for bias—particularly in predictive modeling—cannot be entirely excluded. Future studies with actively balanced cohorts or larger sample sizes are warranted to confirm and extend our observations. Our current implantation model using Ishikawa cells has several limitations. First, due to their malignant origin, their hormonal responses may not fully reflect physiological endometrial regulation (55). Moreover, it lacks the multicellular complexity (stromal, immune cells) critical for *in vivo* implantation. Further validation with animal or organoid models is required. Finally, this study focused on peripheral blood rather than endometrial miRNA expression profiles due to the non-invasive, sensitive, and real-time nature of blood-based sampling. Although endometrial miRNAs may offer better causal interpretability, the blood-based miRNA signature identified here holds potential for predictive models that can be validated in larger populations, ultimately aiding clinical decision-making.

## Conclusions

In conclusion, this research employs the largest sample size to date and is the first to report on the differences in plasma miRNA expression patterns between successful pregnancy and unsuccessful implantation across multiple peri-implantation time points, including D0 (ovulation day), D3, D5, D7, and D9. Not only were dynamic differentially expressed miRNAs identified, but also their distinct expression patterns were described. Associations between these DDE miRNAs with reproductive parameters were evaluated, suggesting their physiological significance. Moreover, we discovered that most target genes of these DDE miRNAs are associated with the embryo implantation process, providing new insights into the molecular mechanisms underlying the pathological process of implantation failure.

## Data Availability

The original data of small RNA sequence generated in the study are publicly available. This data can be found here: Genome Archive (https://ngdc.cncb.ac.cn/gsa-human, GSA-Human: HRA016695). Other data underlying this article will be shared on reasonable request to the corresponding author.

## Supplementary material

Supplementary Figure 1 Overview of the study design. The day of ovulation assessed by a gynecologist was based on plasma LH level and release of the dominant follicle was denoted as Day 0 (D0).

Supplementary Figure 2 Line chart showing the dynamic changes in hormone concentrations during peri-implantation stage between the implantation success and failure groups. **(A)** estradiol (E2) and **(B)** progesterone (P). Time (d) starts from the day of ovulation, e.g. Day 0 (D0) refers to the ovulation day and D9 refers to 9 days after the ovulation day. The significance of *p*-values was labeled as: * 0.01 < *p*-value < 0.05, **** *p*-value < 0.0001.

Supplementary Figure 3 Principal Component Analyses and T-SNE of miRNA sequencing experiments in screening set. **(A)** principal component analysis (PCA) for the total miRNA profile between batches and groups **(B)** before batch effect correction. **(C)** PCA for the total miRNA profile between batches and **(D)** groups after batch effect correction. **(E)** PCA and **(F)** T-SNE for outlier detection.

Supplementary Figure 4 Regulatory miRNA-mRNA networks for dynamic differentially expressed miRNAs belonging to the pattern R to G and pattern T2 to G.

Supplementary Figure 5 Assessment of miRNA expression in the validation set. Six DDE miRNAs are identified as replicative DDE miRNAs as shown in screening set. **(A-F)** show the expressions of DDE miRNAs (hsa-miR-125b-1-3p, hsa-miR-1323, hsa-miR-195-5p, hsa-miR-214-3p, hsa-miR-4433b-3p, and hsa-miR-99b-5p) between implantation failure and success groups. Wilcoxon rank-sum test was used for statistical analysis. *P*-values indicate comparisons between different time points within each group, with distinct colors used to distinguish the groups. *comparisons were performed between implantation failure and success groups (**p-*value<0.05, ***p-*value<0.01, ****p-*value<0.001).
