# Supplementary material for: Temporal trajectory of plasma miRNA during the peri-implantation stage: comparing implantation success and failure in single frozen–thawed blastocyst transfers
Source: Front Endocrinol (Lausanne). 2026 Feb 16;17:1708664. doi: 10.3389/fendo.2026.1708664 (PMC12950558; doi:10.3389/fendo.2026.1708664)
Supplement: Supplementary Figure 1 — Overview of the study design. The day of ovulation assessed by a gynecologist was based on plasma LH level and release of the dominant follicle was denoted as Day 0 (D0). [file SupplementaryFile1.docx]

**Supplementary Figures**

**
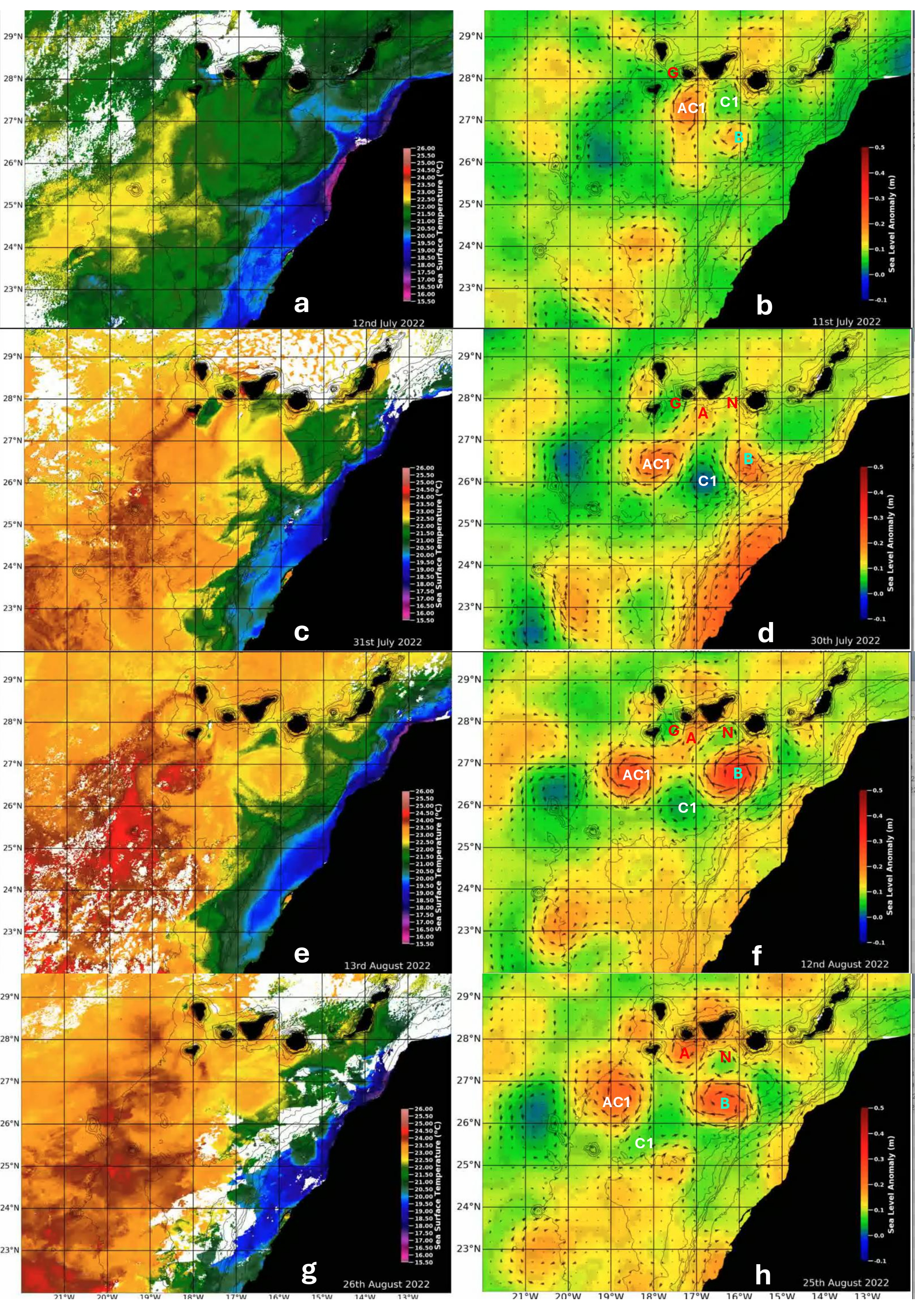
**

**
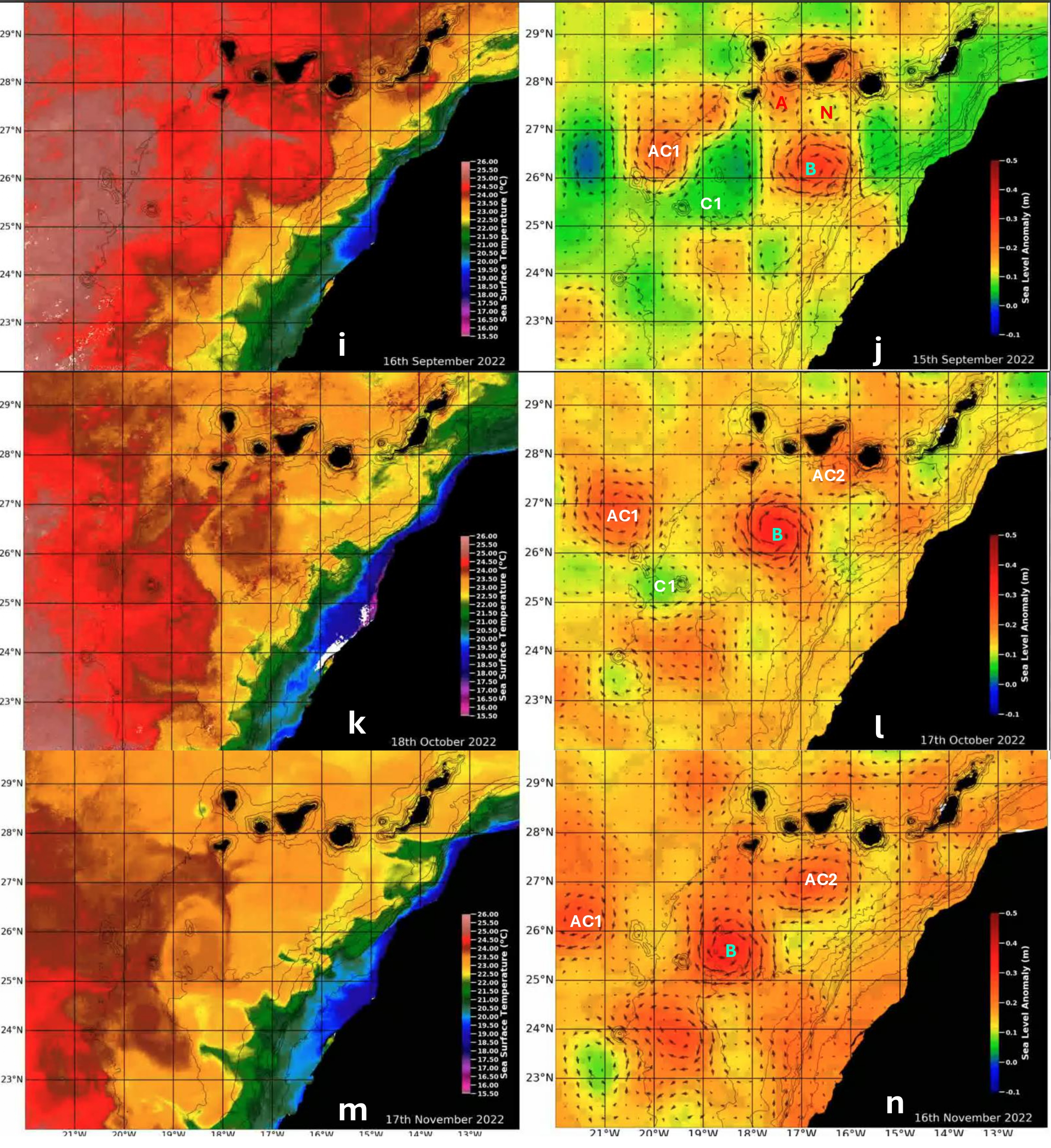
**

**Figure S1:** Tracking of the studied eddies using sea surface temperature (SST, °C) from the Visible Infrared Imaging Radiometer Suite (VIIRS) and sea level anomaly from AVISO.
G: Garajonay; N: Nublo; A: Anaga; B: Bentayga; AC1 and AC2: anticyclonic eddies not sampled; C1: cyclonic eddy not sampled.

G: First detected in SST on 11–12 July (**a–b**), approximately one month before being sampled on 7 August (**e–f**). One month later (**g–h**), the surface SST signal had disappeared.

C1 and AC1: This eddy pair was beginning to detach downstream of the islands when G was first observed (**a–b**) and subsequently drifted along the Canary Eddy Corridor (CEC) during the following months (**c–n**).

N and A: This eddy pair replaced C1–AC1 and began forming in late July, just prior to the start of the cruise (**c–d**). The eddies remained attached to the islands throughout the cruise and until mid-September (approximately 1.5 months), but vanished in October (**k–l**), likely due to merging, as Eddy Bentayga (B) prevented their southward drift.

AC2: This anticyclonic eddy began forming south of Tenerife in mid-October, when A likely detached, merged, and disappeared (**k–l**).

B: Generated south of Gran Canaria approximately five months before being sampled in November 2021 (**a–b**). The life history of this eddy is well described in Valencia et al. (2025) and was clearly tracked in satellite sea level anomaly data over several months.

**Figure S2:** Absolute azimuthal velocity (perpendicular to the transect) at 50 dbar depth, derived from SADCP measurements, is shown along (**A**) BGT2, where green shading indicates regions of lower velocity and brown shading highlights areas of cyclonic vorticity, and (**B**) Bentayga eddy (Stns 11-27), in where, three subregions are identified: (1) a core region (Co) between Stns 15-20, with it center at Stn 18, marked by minimal azimuthal velocity; (2) an inner ring (Ir), extending from Stns 13-15 on the eastern side and 20-23 on the western side, where azimuthal velocity peaks; and (3) an outer ring (Or) surrounding the inner ring, spanning Stns 12-13 and 23-26.

**Figure S3**: Vertical distribution of suspended particulate organic (**A**) carbon (POCsus) and (**B**) nitrogen (PONsus), both in µmol L^-1^. Each box, along the horizontal axis, represents one of the biogeochemical transects (BGT1, BGT2, BGT3; left to right). Colored brackets indicate the presence of eddies—Garajonay (GJ; green), Anaga (AN; red), Nublo (NB; blue), and Bentayga (orange tones). For Bentayga, the core (Co), inner ring (Ir) and outer ring (Or) are distinguished. Pycnoclines are shown as thick black lines; in Bentayga, a dashed line marks the upper pycnocline and the boundary of the low-salinity core; (**C**) Model II relationship between POCsus and PONsus measurements from BGT1 (orange dots), BGT2 (blue dots), and BGT3 (green dots). Outliers from the first (orange triangles) and second (dark blue triangles) cruises are indicated, and the resulting orthogonal distance regression line is shown.

**Figure S4:** Distribution of (**A**) maximum Brünt-Väisälä frequency (in 10^-4^ s^-2^) and (**B**) density (σ S, θ, Z) in kg m^-3^. The 25.6 kg m^-^³ isoline is marked by a thicker white line. Both distributions have a vertical resolution of 1 dbar. Each box, along the horizontal axis, represents one of the biogeochemical transects (BGT1, BGT2, BGT3; left to right). Colored brackets indicate the presence of eddies—Garajonay (GJ; green), Anaga (AN; red), Nublo (NB; blue), and Bentayga (orange tones). For Bentayga, the core (Co), inner ring (Ir) and outer ring (Or) are distinguished. Pycnoclines are shown as thick black lines; in Bentayga, a dashed line marks the upper pycnocline and the boundary of the low-salinity core.

**Figure S5:** (**A, B**) Depth-weighted average (± standard deviation) values of potential temperature (θ) and salinity, in the surface mixed layer (5 dbar to pycnocline; orange bars) and below the pycnocline (pycnocline to 200 dbar; dark blue bars), 1 dbar vertical resolution. Numbers above the θ bars indicate the pycnocline depth (in dbar). Colored squares below the x-axis indicate stations within each eddy: Garajonay (green), Anaga (red), Nublo (blue), and Bentayga, with orange tones distinguishing Bentayga’s core (light orange), inner ring (medium orange) and outer ring (dark orange). An “X” marks the station nearest each eddy center.

**Figure S6:** (**A**) Chlorophyll-*a* fluorescence (FChl-*a*) averaged across five depths above and below the deep chlorophyll maximum (DCM). The DCM depth is indicated by the orange line. Numbers above FChl-*a* bars indicate the DCM depth; (**B**) Depth-weighted average (± standard deviation) values of apparent oxygen utilization (AOU) in the surface mixed layer (5 dbar to pycnocline; orange bars) and below the pycnocline (pycnocline to 200 dbar; dark blue bars). Both have 1 dbar vertical resolution. Colored squares below the x-axis indicate stations within each eddy: Garajonay (green), Anaga (red), Nublo (blue), and Bentayga, with orange tones distinguishing Bentayga’s core (light orange), inner ring (medium orange) and outer ring (dark orange). An “X” marks the station nearest each eddy center.

**Figure S7:** Vertical distributions of (**A**) dissolved inorganic nitrogen (DIN), (**B**) phosphate (PO_4_^3-^) and (**C**) Silicate (SiO_4_^4-^), all in µmol L^-1^. Each box, along the horizontal axis, represents one of the biogeochemical transects (BGT1, BGT2, BGT3; left to right). Colored brackets indicate the presence of eddies—Garajonay (GJ; green), Anaga (AN; red), Nublo (NB; blue), and Bentayga (orange tones). For Bentayga, the core (Co), inner ring (Ir) and outer ring (Or) are distinguished. Pycnoclines are shown as thick black lines; in Bentayga, a dashed line marks the upper pycnocline and the boundary of the low-salinity core.

**Figure S8**: (**A, B**) Depth-weighted average (± standard deviation) concentrations of dissolved organic carbon (DOC) and suspended particulate organic carbon (POCsus), in the surface mixed layer (5 dbar to the pycnocline; orange bars) and below the pycnocline (pycnocline to 200 dbar; dark blue bars) across sampling stations. For POCsus only BGT1 and BGT2 data are represented. Colored squares below the x-axis indicate stations within each eddy: Garajonay (green), Anaga (red), Nublo (blue), and Bentayga, with orange tones distinguishing tones distinguishing Bentayga’s core (light orange), inner ring (medium orange) and outer ring (dark orange). An “X” marks the station nearest each eddy center.

**Figure S9**: (**A, B**) Depth-weighted average (± standard deviation) values of absorption coefficient at 254 nm (a_CDOM_(254)), and at 320 nm (a_CDOM_(320)) in the surface mixed layer (5 dbar to pycnocline; orange bars) and below the pycnocline (pycnocline to 200 dbar; dark blue bars). Colored squares below the x-axis indicate stations within each eddy: Garajonay (green), Anaga (red), Nublo (blue), and Bentayga, with orange tones distinguishing tones distinguishing Bentayga’s core (light orange), inner ring (medium orange) and outer ring (dark orange). An “X” marks the station nearest each eddy center.

**Figure S10:** (**A**, **B**) Depth-weighted average (± standard deviation) values of fluorescence at Ex/Em pair 320/410 nm (Peak M), and at 280/350 nm (Peak T) in the surface mixed layer (5 dbar to pycnocline; orange bars) and below the pycnocline (pycnocline to 200 dbar; dark blue bars). Colored squares below the x-axis indicate stations within each eddy: Garajonay (green), Anaga (red), Nublo (blue), and Bentayga, with orange tones distinguishing tones distinguishing Bentayga’s core (light orange), inner ring (medium orange) and outer ring (dark orange). An “X” marks the station nearest each eddy center.
